# Alkali Release from Aggregates in Long-Service Concrete Structures: Laboratory Test Evaluation and ASR Prediction

**DOI:** 10.3390/ma11081393

**Published:** 2018-08-09

**Authors:** Mario Berra, Teresa Mangialardi, Antonio Evangelista Paolini

**Affiliations:** 1 Ricerca sul Sistema Energetico (RSE S.p.A.), Via R. Rubattino 54, 20134 Milano, Italy; mario.berra@rse-web.it; 2 Department of Chemical Materials Environment Engineering, Faculty of Civil and Industrial Engineering, La Sapienza University, 00184 Roma, Italy; antonioevangelista.paolini@uniroma1.it

**Keywords:** alkali-silica reaction, concrete aggregates, long-term alkali release, alkali extraction test, ASR prediction model

## Abstract

This paper proposes a simple model for predicting the development of deleterious expansion from alkali-silica reaction (ASR) in long-service concrete structures. This model is based on some composition and reactivity parameters related to ASR, including the long-term alkali contribution by aggregates to concrete structures. This alkali contribution was estimated by means of a laboratory extraction test, appositely developed in this study in order to maximize the alkali extraction within relatively short testing times and with low leaching solution/aggregate ratios. The proposed test is a modification of the Italian Standard test method UNI 11417-2 (Ente Nazionale Italiano di Normazione) and it consists of subjecting an aggregate sample to leaching with saturated calcium hydroxide solution in a laboratory autoclave at 105 °C. Nine natural ASR-susceptible aggregates (seven sands and two coarse aggregates) were tested and the following optimized test conditions were found: leaching solution/aggregate weight ratio = 0.6; solid calcium hydroxide/aggregate weight ratio = 0.05; test duration = 120 h. The results of the optimized alkali extraction tests were used in the proposed model for predicting the potential development of long-term ASR expansion in concrete dams. ASR predictions congruent with both the field experience and the ASR prevention criteria recommended by European Committee for Standardization Technical Report CEN/TR 16349:2012 were found, thus indicating the suitability of the proposed model.

## 1. Introduction

Deleterious expansions associated to Alkali-Silica Reaction (ASR) have been repeatedly reported in the last decades among the main causes of deterioration of concrete structures [1,2].

ASR is a slowly expansive reaction between certain forms of alkali-reactive silica (opaline silica, flint, and cryptocrystalline quartz) and/or certain silicate minerals present in concrete aggregates and the hydroxyl ions in concrete pore solution, mainly associated with the alkaline metal ions (sodium and potassium) [3,4,5]. This reaction leads to the formation of an alkali-silicate gel, which absorbs water and swells, causing internal expansive pressure [6,7,8].

Most of the alkalis are available in the concrete since the construction phase of the structures and come primarily from the cement, with eventual minimal contributions from the mix water and the chemical admixtures. However, enhancement of the alkali concentration in concrete may arise during the service life of concrete structures when aggregates with alkali-bearing minerals (i.e., volcanic glasses, feldspars, micas, clay minerals, nepheline, and zeolites) release their alkalis into the concrete pore solution, over a long time [9,10,11,12].

The concrete alkali enrichment is a consequence of the alkaline cations release from aggregates. In order to maintain the electroneutrality of the pore solution, it is assumed that the hydroxide ion concentration increases as well [13], thus triggering and/or accelerating ASR expansion development, even if the initial alkali content of the concrete is insufficient to promote ASR expansion development [10].

This chemical mechanism is generally accepted to explain the observed cases of discrepancy between measured and expected alkali contents in field concrete structures showing late or progressive ASR-induced distress, such as, for example, the case of concrete dams, in consideration of their very high content of aggregates and their required long service life. In concrete dams containing alkali-reactive aggregates, deleterious ASR expansion may occur long time after construction (initiation phase of more than 20 years) even if low-alkali Portland cement is used as an ASR preventive measure [14]. Slowly increasing expansions together with resulting distress may be still observed over a very long timescale, even 40 year after construction (undefined propagation phase) [15,16]. This expansive phenomenon may gradually reduce the serviceability and even the load carrying capacity and safety of the concrete structures, with high cost of repairs and replacements [17,18,19,20,21].

However, based on their own experimental results, some authors [22] recently questioned the existence of a relationship between alkali metal enrichment and hydroxide ion concentration increase in the concrete pore solution.

Enhancement of the alkali concentration in concrete may also arise during the service life of concrete structures when they are exposed to external sources of potential alkalis (for example, deicing salts in the case of concrete pavements and bridges) [23,24,25].

Differently from the alkali release from concrete aggregates, the alkali contribution from external sources concerns only some concrete structures and it is mainly localized at structure surface layers of some components (e.g., bridge decks). Moreover, it is very difficult to quantify this contribution in terms of increased concrete alkali content, also due to surface leaching phenomena [26]. Therefore, long-service concrete structures not exposed to external alkali sources were only considered in the present study.

For such structures, the knowledge of the amount of alkalis releasable from aggregates represents an issue of particular concern in developing models aimed at predicting eventual long-term deleterious ASR expansion of mass concrete.

Considering that the determination of the alkali release from aggregates in the field conditions is very difficult, laboratory test methods have been proposed in the literature for estimating the long-term alkali contribution by aggregates to concrete structures.

These tests are directly carried out on concrete aggregates, by using caustic solutions such as saturated calcium hydroxide solution or 0.7 M NaOH and 0.7 M KOH solutions, as extraction media. The use of saturated calcium hydroxide solution at boiling temperature is specified by the Italian standard test method UNI 11417-2 (test duration 6 h) [27] and the French LPC 37 test method (test duration 7 h) [28]. The use of 0.7 M NaOH and 0.7 M KOH solutions at relatively low temperatures (38 °C or 60 °C) was investigated by several researchers [10,29,30,31] for contact times varying between 180 and 580 days. The test with NaOH and KOH solutions with solid lime in excess is currently under investigation and validation by RILEM (International Union of Laboratories and Experts in Construction Materials, Systems and Structures) [32]. The role played by calcium hydroxide in extracting alkalis from aggregates is of great importance. Indeed, sodium and potassium hydroxides alone can give rise to ion exchanges between the solid phase and the aqueous solution placed in contact without increasing the pH or hydroxide ion concentration. On the contrary, the presence of calcium ions in this solution produces Ca–Na and Ca–K ion exchanges able to enrich the solution with Na^+^ and K^+^ ions, and solid calcium hydroxide dissolve, thus supplying OH^-^ ions to maintain the solubility equilibrium and electroneutrality with the alkali ions. This ion exchange with Ca^+2^ can potentially increase the alkali leaching [29].

The above-mentioned leaching tests provide different results, depending on the aggregate fineness, the solution adopted, the operating conditions (solution/aggregate and calcium hydroxide/aggregate ratios, temperature and testing times etc.). If carried out at the same temperature, saturated calcium hydroxide solution is much less aggressive compared to sodium and potassium hydroxide solutions, due to a lower pH of the solution and the formation of reaction products able to incorporate some of the released alkali ions [10,30]. However, being generally performed at the boiling temperature, leaching tests with saturated calcium hydroxide solutions are very rapid compared to the time consuming leaching tests based on sodium and potassium hydroxide solutions performed at 38 °C or 60 °C.

Presently, alkali releases obtained through all laboratory tests proposed in the literature still need to be verified with those in field conditions, and procedures for this verification need to be developed. By the way, this is within the present plan of activity of RILEM TC 258 AAA.

In the present study, in order to maximize the alkali release from concrete aggregates within relatively short testing times and with low leaching solution/aggregate ratios, an appositely modified version of the current Italian UNI standard test method was developed using nine natural ASR-susceptible aggregates (seven sands and two coarse aggregates) with different petrographic and alkali-reactivity characteristics.

Moreover, a simple model was developed to predict the potential effect of alkali release from the aggregates investigated on the development of deleterious ASR expansion in long-service structures, such as concrete dams. In this model, the results of the proposed alkali extraction test were used together with some parameters that are characteristic of alkali-silica reaction and related to the composition of concrete.

## 2. A Simple Model for Evaluating the Effect of Alkali Release from Aggregates on Deleterious ASR Expansion Development

The proposed model was based on the knowledge of four key parameters: (1) the initial alkali content of the concrete mix used for the structure construction, $Lac_{0}$; (2) the Threshold Alkali Level, TAL, of the aggregate used in the concrete mix; (3) the long-term alkali contribution by this aggregate to concrete mix, $Lac_{\infty }^{agg}$; and (4) the efficacy parameter, $R_{max}$, related to the cement used in concrete mix.

As reported in our previous papers [33,34], the Threshold Alkali Level is an appropriate reactivity parameter for assessing the alkali-reactivity of concrete aggregates. The aggregate TAL (kg Na_2_Oeq/m^3^) is defined as the minimum alkali content of a standard concrete mix made with Portland cement and the aggregate under examination, above which deleterious expansion of concrete will occur. The higher is the aggregate reactivity, the lower the TAL value. The aggregate TAL can be determined through laboratory accelerated expansion tests using standard concrete mixes with different alkali contents [33].

The efficacy parameter $R_{max}$ (kg Na_2_Oeq/m^3^) is defined as the maximum alkali consumption by a blended cement in correspondence of the maximum concrete alkali content, $Lac^{*}$, that does not produce deleterious ASR expansion [35,36]. This efficacy parameter is related to the adsorption/incorporation of alkalis by the hydration products of the cement. In the case of using Portland cement, $R_{max}$ is taken equal to zero. For blended cements containing active mineral additions such as granulated blast furnace slag, coal fly ash, or natural pozzolan, $R_{max}$ is above zero. The parameter $R_{max}$ depends on the type, properties and amount of active mineral addition of blended cements [37,38] and can be determined for each blended cement through laboratory expansion tests on concrete mixes of standard composition made with the cement under examination, an aggregate with known TAL value and different alkali contents [36].

In concrete structures containing ASR-susceptible aggregates, deleterious expansion will occur when the amount of alkali-silicate gel formed by alkali-silica reaction and the concomitant water absorption by this gel are sufficient to develop deleterious swelling pressures.

Based on the TAL and $R_{max}$ definitions, the maximum concrete alkali content $Lac^{*}$ (kg Na_2_Oeq/m^3^) not producing deleterious ASR expansion in concrete can be expressed as:

(1) $$Lac^{*}=TAL+\;R_{max}$$

where TAL and $R_{max}$ are as previously defined.

The concrete alkali content, Lac (kg Na_2_Oeq/m^3^), related to the alkali contributions by the various concrete mix components (cement, admixtures, and aggregates) can be expressed as:

(2) $$Lac=\;Lac_{0}+Lac_{\infty }^{agg}$$

where $Lac_{0}$ (kg Na_2_Oeq/m^3^) is the alkali content of the concrete mix at the time of structure construction (initial concrete alkali content) and $Lac_{\infty }^{agg}$(kg Na_2_Oeq/m^3^) is the long-term alkali contribution from the aggregates.

The value of $Lac_{0}$ is calculated from the concrete mix composition by assuming a total release of alkalis from Portland or blended cement and eventual chemical admixtures and no alkali release from the aggregates.

As anticipated, the value of $Lac_{\infty }^{agg}$ may not be determined in the field conditions. In this study, the long-term alkali contribution from aggregates was assumed to be equal to the alkali contribution, $Lac_{lab}^{agg},$ evaluated from laboratory extraction tests under optimized conditions, established as described at Section 4.1.1. Therefore, $Lac_{\infty }^{agg}$ was replaced by $Lac_{lab}^{agg}$ in Equation (2).

According to Equations (1) and (2), deleterious ASR expansion in concrete will develop only if the following condition is satisfied:

(3) $$Lac>\;Lac^{*}.$$

Combining Equations (1) and (3) yields:

(4) $$Lac>\;TAL+\;R_{max}.$$

Equation (4) holds for blended cements containing active mineral additions ($R_{max}$ > 0), while this equation reduces to Lac>TAL for concrete mixes made with Portland cements ($R_{max}=0$).

Therefore, the value of $R_{max}$ represents a measure of the capability of a specific blended cement in counteracting ASR expansion development in concrete. Values of $R_{max}$ equal to 3.80, 5.50, and 8.10 kg Na_2_Oeq/m^3^ have been reported [39] for some commercial cements, i.e., a blast-furnace slag cement (CEM III/B), a coal fly ash cement (CEM IV/A-V) and a natural pozzolan cement (CEM IV/B-P), respectively. The cement nomenclature is in accordance to European Standard EN 197-1 [40].

It follows that the development of deleterious ASR expansion in long-service concrete structures will be mainly dependent on the composition of the concrete mix ($Lac_{0}$), the type of cement ($R_{max}$) and the alkali-reactivity of aggregate (TAL) used in the concrete mix.

It is evident that, irrespective of the type of cement (Portland or blended cement) and the long-term alkali contribution by aggregates ($Lac_{\infty }^{agg}$), the risk of deleterious ASR development will be irrelevant if aggregates with high TAL values and concrete mixes with relatively low $Lac_{0}$ values are selected.

On the other hand, if aggregates with relatively low TAL values are used (that is increasingly frequent in the concrete construction industry) together with Portland cements, the long-term alkali contribution by aggregates ($Lac_{\infty }^{agg}$) could be very critical in promoting the long-term deleterious ASR expansion development.

In this regard, for concrete mixes designed with given $Lac_{0}$ values, combination of Equations (2) and (4) allows the calculation of the maximum long-term alkali contribution from aggregates, $Lac_{\infty }^{agg},$ for which no deleterious ASR expansion will develop in concrete (maximum tolerable alkali contribution). Conversely, if the long-term alkali contribution from aggregates is known (i.e., evaluated by laboratory extraction tests), the above two equations may be used to calculate the maximum $Lac_{0}$ value that will not produce long-term deleterious ASR expansion (maximum tolerable $Lac_{0}$). On the basis of the cement content of the concrete and the maximum tolerable $Lac_{0}$ value, it is also possible to calculate the maximum tolerable alkali content of the cement, in terms of percentage Na_2_Oeq.

## 3. Materials and Methods

### 3.1. Aggregates Tested

Seven natural sand-sized aggregates were tested in this study: four sands came from Italian quarries (referred to as S1–S4), the other three sands, known for their high alkali contents, came from Norway, Portugal and Spain (labelled as S5, S6, and S7, respectively). Table 1 gives the lithological composition of these sands.

Two coarse aggregates (size gradation 12–32 mm), coming from the same quarries of sands S1 and S2 and exhibiting similar lithological composition as the respective sands, were also tested and designated as aggregates C1 and C2, respectively.

According to the RILEM AAR-1 petrographic method [41], all the aggregates investigated were classified as Class III-S (very likely to be alkali-silica reactive).

Each aggregate was analyzed for the contents of sodium and potassium by the following test procedure: (1) grinding of the aggregate sample to fineness below 45 μm; (2) alkaline fusion of a powdered aggregate-lithium metaborate mixture (weight ratio 1:6) in platinum crucible at a temperature of 1000–1200 °C; (3) quenching of the melt with water and dissolution of the solid with 10% HNO_3_ solution; (4) appropriate dilution of the resulting solution with deionized water and determination of the sodium and potassium ion concentrations by flame Atomic Absorption Spectrophotometer (AAS) according to the procedure reported in [42].

Table 2 gives the contents of sodium and potassium of each aggregate, both in terms of g alkaline metal (Na, K)/kg dry aggregate and in terms of g alkalis (Na_2_O, K_2_O, Na_2_Oeq)/kg dry aggregate.

Prior to leaching tests (extraction of alkalis from aggregates), all the aggregates were characterized for their grain size distribution by dry sieving. The two coarse aggregates were used as received, while the different sands were used with the same grain size gradation that was obtained by appropriate recombination of the various grain size fractions.

Table 3 and Table 4 give the grain size gradations of the recombined sands and the coarse aggregates, respectively.

### 3.2. Leaching Test Procedure

As anticipated, the release of alkalis from aggregates was evaluated by using the extraction test method reported in the Annex A of the Italian Standard UNI 11417-2 [27], both in its current version and in a modified version, appositely developed in the present study.

The current UNI 11417-2 test method consists of subjecting the aggregate sample under examination to a solubilizing attack by reflux with a saturated calcium hydroxide solution for a contact time, *t_c_*, of 6 h, at the boiling temperature. The weight ratio between the saturated Ca(OH)_2_ solution (leachant) and the dry aggregate sample (L/S ratio) is equal to 0.6 g leachant/g dry aggregate and the weight ratio between solid calcium hydroxide (CH) and dry aggregate (CH/S ratio) is equal to 0.05 (5 g CH/100 g dry aggregate). The solubilizing attack is due to the OH^-^ ions action on the siloxane and silanol groups of the reactive silica contained in the aggregate and to the subsequent ion exchange between alkaline metals (Na and K) and calcium ions arising from calcium hydroxide with formation of NaOH and KOH (base exchange) [29]. Therefore, this process is commonly referred to as extraction (or release) of alkalis from aggregates.

**Figure 1:**
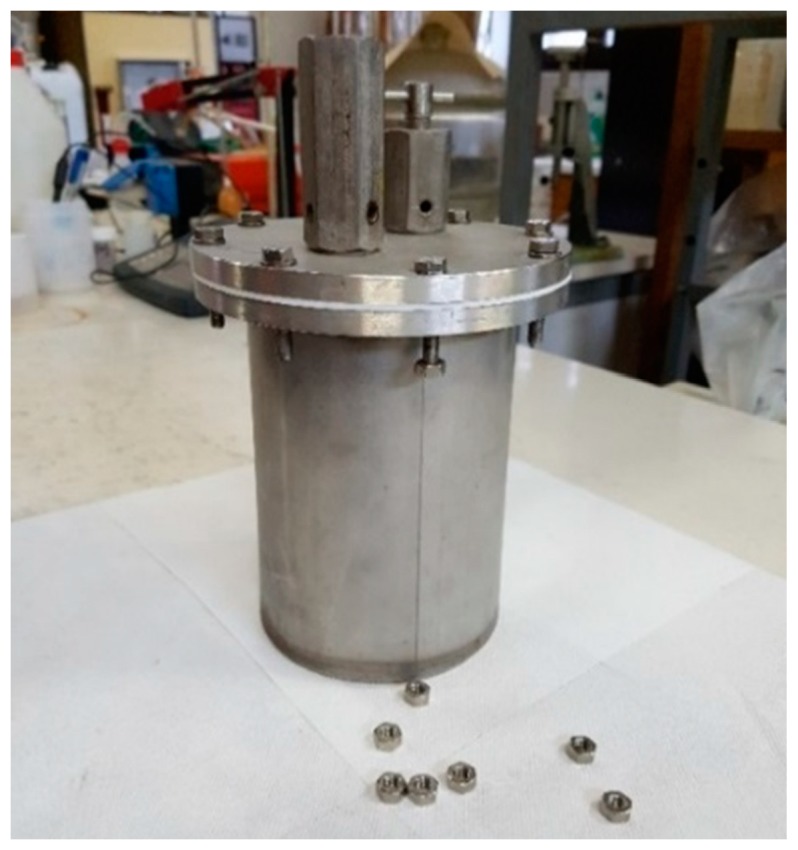
Laboratory autoclave for alkali extraction tests from aggregates.

In the modified version of the UNI test method, the glass boiler equipped with the reflux condenser was replaced by a laboratory autoclave having a volumetric capacity of 1.3 L (Figure 1) placed in a laboratory oven operating at a temperature of 105 °C. Moreover, the amount of aggregate subjected to leaching tests was reduced from 400 g (standard test method) to 200 g. With the use of this autoclave it was possible to greatly prolong the test duration beyond six hours, in order to maximize the alkali release from aggregates.

Using the two different equipment (glass boiler or autoclave) and the different amounts of aggregate, leaching tests on sand S1 were first performed under the test conditions, specified in the UNI standard test method.

The main reasons for the choice of testing sand S1 were the great availability of this material in both fine and coarse grain size gradations and its known relatively high alkali-reactivity in field conditions.

Successively, leaching tests with the above test modifications were performed on sand S1 by changing the CH/S ratio from 5 to 16 g CH/100 g dry aggregate, the L/S ratio from 0.6 to 3.3 g leachant/g dry aggregate and the contact time, *t_c_*, over the range from 6 to 240 h.

At the end of each leaching test, the autoclaved sample was rapidly cooled, filtered on 0.45 μm paper filter and the solid residue was washed with deionized water in order to quantitatively remove the leachate. The resulting solution (filtrate + washing) was acidified and then analyzed for the concentrations of sodium and potassium ions by AAS. In some cases, pH measurements were also performed on filtrate solutions and blank samples (i.e., samples obtained from tests performed under the same conditions but without aggregate specimens).

Finally, under the optimized CH/S and L/S ratio conditions, leaching tests at different *t_c_* values were also performed on the other aggregates investigated in order to better characterize their alkaline metal releases.

In the case of coarse aggregates C1 and C2, due to their much lower specific surface area as compared to the respective sands, the release of sodium and potassium was evaluated only at the ultimate testing time investigated (240 h). Moreover, in order to guarantee the complete immersion of coarser grains of the aggregates in the leachant solution, the L/S ratio was equal to 3.3 g leachant/g dry aggregate, corresponding to the upper value of the L/S ratio range investigated for sands. The CH/S ratio was kept equal to 5 g CH/100 g dry aggregate.

For each aggregate and test condition considered, three replicate tests were performed and the results obtained were averaged. For each test specimen, the average value was obtained from measurements on at least three solution sampling, by discarding results giving a coefficient of variation higher than 10%.

## 4. Results and Discussion

### 4.1. Optimization of the Leaching Test for the Evaluation of Alkali Release from Aggregates

#### 4.1.1. Alkali Releases from Sands

The results of preliminary leaching tests on sand S1 showed that the release of sodium and potassium determined according the UNI standard test method (use of a glass boiler with reflux; amount of aggregate tested = 400 g) or the modified version of the UNI standard test method (use of autoclave; amount of aggregate tested = 200 g) were similar where the same operating conditions (L/S ratio, CH/S ratio, *t_c_*) were adopted. The releases of sodium or potassium, expressed in terms of mg metal released/kg dry aggregate, $M_{r}$, were equal to: 10.8 mg Na/kg dry aggregate and 12.5 mg K/kg dry aggregate (standard test); 12.9 mg Na/kg dry aggregate and 13.9 mg K/kg dry aggregate (modified test).

**Figure 2:**
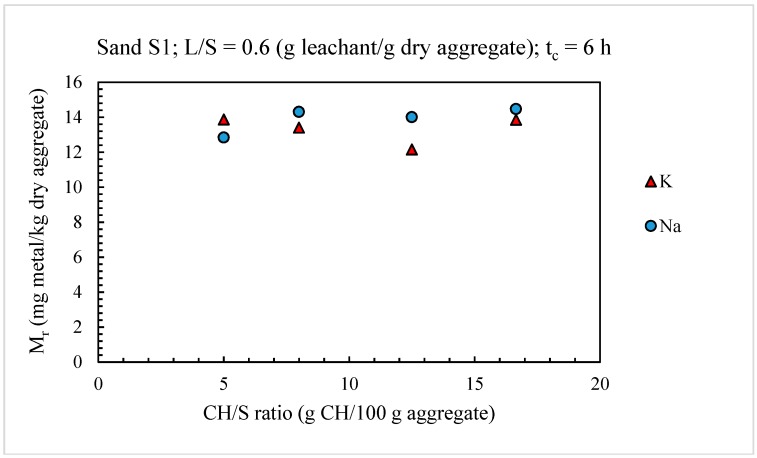
Effect of solid calcium hydroxide (CH)–to-dry aggregate (S) ratio (CH/S) on alkaline metal release from sand S1.

Figure 2 shows the effect of increasing the CH/S ratio on the release, $M_{r}$, of sodium and potassium from sand S1 when the modified test method was used and the values of the contact time, *t_c_*, and L/S ratio were identical to those adopted by the UNI Standard test method (*t_c_* = 6 h; L/S = 0.6 g leachant/g dry aggregate).

These results indicated that the CH/S ratio did not significantly affect the alkaline metal release from the aggregate (percent variations of *M_r_* lower than about 12% for both sodium and potassium), at least within the range of CH/S values considered (5–16 g CH/100 dry aggregate). This was because the amount of solid CH used in the leaching test was largely in excess of the quantity needed to maintain the lime saturation condition in the leaching solution throughout the test.

Therefore, the CH/S ratio of 5 g CH/100 g dry aggregate was maintained in the modified version of the UNI standard test method.

**Figure 3:**
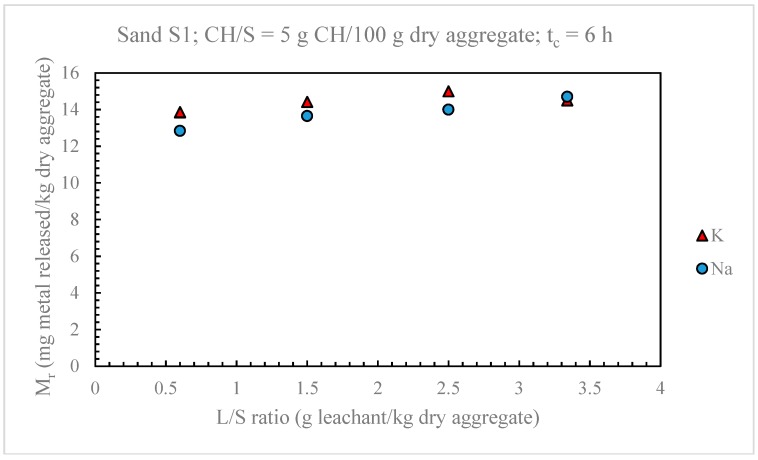
Effect of saturated Ca(OH)_2_ solution (leachant) –to-dry aggregate (S) ratio (L/S) on alkaline metal release from sand S1.

As shown in Figure 3, the release of sodium and potassium was only slightly increasing with the L/S ratio when the leaching tests were carried out using the same values of *t_c_* (6 h) and CH/S ratio (5 g CH/ 100 g dry aggregate) of the UNI Standard test method. Maximum percentage variations of about 14% for sodium and 8% for potassium were observed.

**Figure 4:**
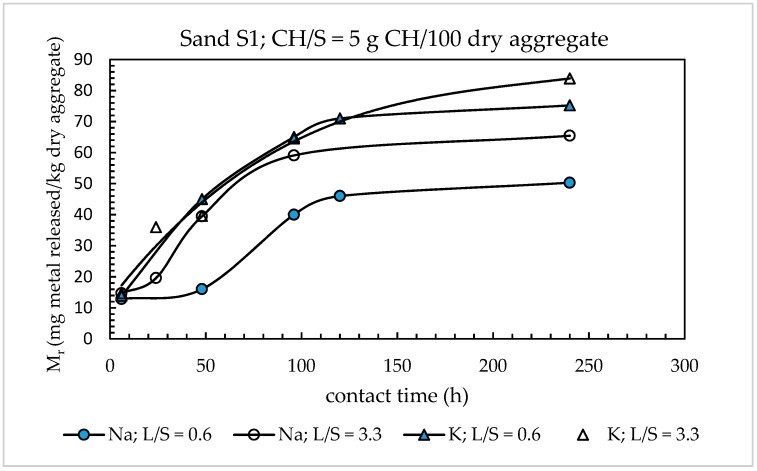
Effect of contact time on alkaline metal release from sand S1 at two different L/S ratios.

In contrast, the release of the two alkaline metals was found to greatly increase with increasing contact time, *t_c_*, irrespective of the L/S ratio used. This effect is highlighted by Figure 4 where the $M_{r}$–*t_c_* curves for K and Na are reported for two different L/S ratios (0.6 and 3.3 g leachant/g dry aggregate) and the same CH/S ratio (5 g CH/100 g dry aggregate). Exception made for the series (K; L/S = 3.3), for which the points were interpolated with a polynomial equation by the method of least squares (R^2^ = 0.9782), all the curves in this figure were drawn joining experimental points. For both sodium and potassium, the $M_{r}$–*t_c_* curves exhibited an asymptotic trend. Values of $M_{r}$ greater than 90% of those achieved at the ultimate testing time investigated (240 h) were attained after 120 h of testing.

As expected, at long contact times (120–240 h), the $M_{r}$ values obtained for both sodium and potassium with the two L/S ratios differed more significantly than the respective values determined at shorter contact times. At 240 h, an increase of the L/S ratio from 0.6 to 3.3 g leachant/g dry aggregate produced a percentage $M_{r}$ increase of 30% for sodium and 14% for potassium, against 15% for Na and 6% for K at *t_c_* = 6 h.

Based on the results of Figure 4, and also considering that: (1) the L/S ratio in real concrete structures is much lower than 0.6 g leachant/g dry solid and (2) that the laboratory tests with L/S ratios of less than 0.6 could pose operational difficulties, the L/S ratio of the UNI standard test method (L/S = 0.6 g leachant/g dry solid) was maintained in the modified version of the alkali extraction test.

Using the values of CH/S = 5 g CH/100 g dry aggregate, and L/S = 0.6 g leachant/kg dry aggregate of the UNI standard test method, leaching tests were performed on the other six sands by varying the contact time, *t_c_*, in order to evaluate the alkaline metal releases from these sands and, at the same time, to confirm the asymptotic trend of the $M_{r}$–*t_c_* curves.

**Figure 5:**
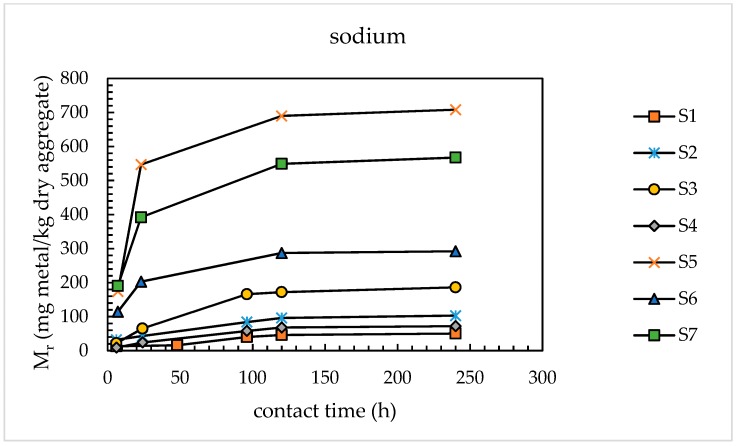
Effect of contact time on sodium release from sands S1–S7.

**Figure 6:**
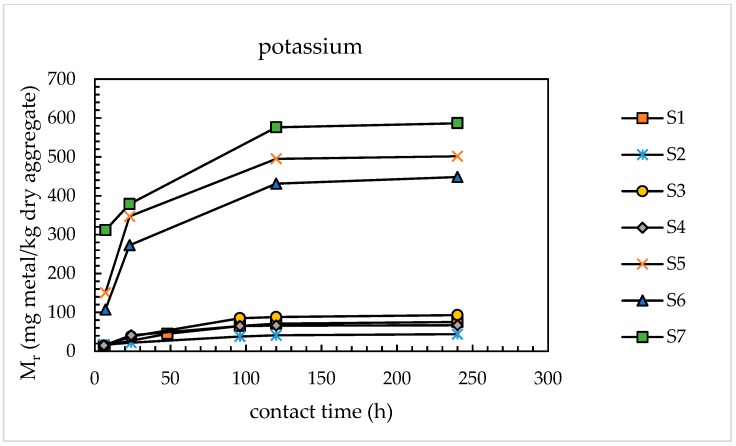
Effect of contact time on potassium release from sands S1–S7.

Figure 5 and Figure 6 show, respectively, the $M_{r}$–*t_c_* curves obtained for sodium and potassium relevant to sands S2–S7. For comparison, the results relevant to sand S1 (same data reported in Figure 4) are re-proposed.

The results in Figure 5 and Figure 6 confirmed the asymptotic trend of the $M_{r}$–*t_c_* curves for all the sands investigated. The $M_{r}$ values determined after 120 h for both alkaline metals were 92–98% of the respective values determined after 240 h. Therefore, it was possible to significantly reduce the test duration with respect to the ultimate testing time investigated. So, as an optimal contact time, a contact time of 120 h was selected in the modified version of the alkali extraction tests. The selection of this time also arose from the experimental evidence of the slurry stiffening when some sands (particularly, sands S3 and S4) were tested until 240 h. This phenomenon greatly hindered the quantitative recovery of the leachate from the autoclaved sample.

Therefore, apart from the use of an autoclave as extraction reactor (in place of a glass boiler with reflux) and the reduction of the amount of aggregate tested (from 400 g to 200 g), the only substantial modification of the operating parameters of UNI standard test method consisted of prolonging significantly the duration of the extraction test from 6 h to 120 h.

Table 5 summarizes the $M_{r}$ values of sodium and potassium determined for all the sands investigated under the optimal test conditions (CH/S = 5 g CH/100 g dry aggregate; L/S = 0.6 g leachant/g dry aggregate; *t_c_* = 120 h). In this table, the percentage release (wt %) of sodium and potassium and the alkali release, ${M^{\prime }}_{r}$, expressed in terms of mg Na_2_O, K_2_O, or Na_2_Oeq/kg dry aggregate are also reported.

The results in Table 5 revealed that, in terms of percentage metal release, the releases of sodium from the sands S1–S4 (0.93–2.90%) were similar to those determined for sands coming from other countries, S5–S7 (1.29–2.84%). In contrast, the release of potassium from sands S1–S4 (0.41–1.47% for potassium) were much lower than those determined for sands S5–S7 (1.00–3.35%). Due to the great differences in alkaline metal contents of sands from different origin (Table 2), sands S5–S7 exhibited values of $M_{r}$ (mg metal/kg dry aggregate) four–six times higher than those determined for sands S1–S4. The overall results of this study were congruent with those reported in the literature by other researchers for alkali extraction tests with sodium and potassium hydroxide solutions as leaching media [29,30,31].

In order to confirm the assumed mechanism of alkali release reported in the Introduction section, pH measurements were performed on filtrate solutions obtained for sands S1 and S5, whose relatively high reactivity in the field was known. The measurements performed at different contact times between solid specimens and saturated calcium hydroxide solution revealed negligible changes in the pH values (pH = 12.16–12.36) in comparison to blank samples (pH = 12.22–12.32). Due to the alkali reactivity of these sands, a reduction of pH would have been expected with increasing testing time. Therefore, the results obtained appeared to be consistent with the hypothesis of a progressive replacement of OH^−^ ions consumed by ASR, through lime dissolution, balancing alkaline metals released by aggregates.

#### 4.1.2. Alkali Releases from Coarse Aggregates

Table 6 gives the releases of sodium and potassium, $M_{r}$ (mg alkaline metal/kg dry aggregate), determined for the coarse aggregates C1 and C2 under the following test conditions (CH/S ratio = 5 g CH/100 g dry aggregate; L/S ratio = 3.3 g leachant/g dry aggregate; *t_c_* = 240 h). In this table, the percentage releases of sodium and potassium and the alkali releases (${M^{\prime }}_{r}$) expressed in terms of mg alkaline metal oxide/kg dry aggregate are also reported.

As expected, in spite of the higher L/S ratio and the longer contact time used for testing coarse aggregates, the percentage releases of sodium and potassium from the aggregates C1 and C2 (0.05 and 0.22% for sodium; 0.11 and 0.16% for potassium) were found to be much lower than those obtained for the respective sands (0.93 and 0.71% for sodium; 1.43 and 0.41% for potassium).

Comparing the data in Table 5 and Table 6, the ratios between the alkali releases (mg Na_2_Oeq/kg dry aggregate) from coarse aggregates and sands coming from the same source ranged from 0.13 to 0.14.

These results show how important is the role of the specific surface area of aggregate in the releasing process by attack of the saturated calcium hydroxide solution, as already pointed out in the literature [13].

Although the release of alkalis from coarse aggregate is very low, this release cannot be neglected in calculating the alkali contribution by combined aggregate (sand + coarse aggregate) to concrete mix. This is because of the high combined aggregate content in the concrete mixes (1800–2100 kg/m^3^ depending on the type of structure) and the high percentage of the coarse aggregate in the combined aggregates (55–65% by weight).

If the release of the coarse aggregate is unknown, it is possible to estimate its alkali contribution to concrete in terms of an equivalent percentage increase of the sand content, $\Delta X_{S}\%$, the latter being calculated on the basis of the following equations:

(5) $$\;M_{r\;Comb}=\;X_{S}M_{rS}+X_{C}M_{rC}$$

with:

(6) $$X_{S}+X_{C}=1$$

where $M_{r\;Comb}$ is the alkaline metal release (Na or K) from the combined aggregate, $X_{S}$ is the weigh fraction of sand in the combined aggregate, $X_{C}$ is the weigh fraction of coarse aggregate in the combined aggregate, $M_{rS}$ and $M_{rC}$ are the alkaline metal releases from sand and coarse aggregate, respectively.

The increased weight fraction of sand, ${X^{\prime }}_{S}$, is given by:

(7) $${X^{\prime }}_{S}=M_{r\;Comb}/M_{rS}.$$

Combining Equations (5)–(7) yields:

(8) $${X^{\prime }}_{S}=X_{S}+\;\frac{\left(1-X_{S}\right)M_{rC}}{M_{rS}}.$$

The percentage increase of sand content $\Delta X_{S}\%$ is calculated as:

(9) $$\Delta X_{S}\%=\frac{\left(X_{S}^{\prime }-X_{S}\right)}{X_{S}}\cdot 100,$$

and the estimated alkaline metal release from the combined aggregate, $M_{r\;Comb}^{*}$, can be calculated as:

(10) $$M_{r\;Comb}^{*}=\;\left(1+\frac{\Delta X_{S}\%}{100}\right)X_{S}M_{rS}.$$

Using the $M_{r}$ values in Table 5 ($M_{rS}$) and Table 6 ($M_{rC}$) and Equations (5)–(9), the $\Delta X_{S}\%$ values were calculated for the combinations S1-C1 and S2-C2 for $X_{S}$ equal to 0.35 or 0.45. The $\Delta X_{S}\%$ values thus calculated are reported in Table 7.

As can be noted from Table 7, the $\Delta X_{S}\%$ values depended on both the type of alkaline metal and the sand-coarse aggregate combination considered. The $\Delta X_{S}\%$ values ranged from 14.4 to 24.2% for sodium releases and from 18.9 to 36.1% for potassium releases. An overall average $\Delta X_{S}\%$ value of 23.3% was calculated.

Using the average $\Delta X_{S}\%$ value and the $M_{rS}$ values reported in Table 5, the estimated values of $M_{r\;Comb}$ for sodium and potassium, indicated as $M_{r\;Comb}^{*}\;,$ were calculated with Equation (10) and compared in Table 7 with the respective $M_{r\;Comb}$ values.

The values of $M_{r\;Comb}$ and $M_{r\;Comb}^{*}$ were found in good agreement, their maximum percentage difference being equal to 11.5%. Therefore, a $\Delta X_{S}\%$ value of 23% may be used for estimating the alkali contribution by coarse aggregate to concrete mix, in terms of equivalent sand content.

### 4.2. Application of the Proposed Model for Evaluating the Effect of Alkali Release from Aggregates on Deleterious ASR Development in Long-Service Structures

The model described in Section 2 was applied to predict the potential effect of alkali release from the aggregates investigated in this study on the development of ASR expansion in concrete dams.

A typical concrete mix composition for concrete dams is as follows: cement content = 200 kg/m^3^; water content = 120 kg/m^3^; aggregate content = 2100 kg/m^3^, of which 35% as sand and 65% as coarse aggregate. Use of two types of cement was considered: a Portland cement (CEM I) with Na_2_Oeq = 1.0% and a pozzolanic cement made with natural pozzolan (CEM IV/B-P) with Na_2_Oeq = 2.20% and $R_{max}$ = 8.10 kg Na_2_Oeq/m^3^ [40].

Based on the assumed concrete composition, the $Lac_{0}$ values were equal to 2.0 kg Na_2_Oeq/m^3^ for Portland cement concrete and 4.4 kg Na_2_Oeq/m^3^ for pozzolanic cement concrete.

An alkali release from coarse aggregate equivalent to that resulting from an increase of the sand content equal to 23% by weight (Section 4.1.2) was considered.

Using the values of alkali releases from each sand determined by laboratory extraction tests and expressed in terms of g Na_2_Oeq/kg dry aggregate (Table 5) and the above concrete mix composition, the $Lac_{lab}^{agg}$ (kg Na_2_Oeq/m^3^) values were calculated for each combined aggregate.

In Table 8, the values of $Lac_{lab}^{agg}$ are reported together with the TAL values of the aggregates investigated and the Lac values calculated with Equation (1) for concrete mixes made with Portland cements ($R_{max}$ = 0).

The TAL values of aggregates 1 and 2 were available in [36], those of aggregates 3 and 4 in [33] while the TAL of aggregate 5 was calculated from the data reported in [43]. No information was available about the TAL values of aggregates 6 and 7.

As expected, the $Lac_{lab}^{agg}$ values for the four aggregates coming from Italian quarries (0.10–0.26 kg Na_2_Oeq/m^3^) were much lower than those obtained for the three aggregates coming from other countries (0.62–1.12 kg Na_2_Oeq/m^3^). This was related to the lower initial alkali content of sands S1–S4 (1.07–2.62% as Na_2_Oeq), compared to sands S5–S7 (4.44–6.41% as Na_2_Oeq) (Table 2).

With respect to the $Lac_{0}$ value of the considered Portland cement concrete mix (2.0 kg Na_2_Oeq/m^3^), the significance of such contributions was very different: marginal significance for concretes made with aggregates 1–4 (percentage $Lac_{0}$ increases of 5–13%), great relevance for concretes containing aggregates 5–7 (percentage $Lac_{0}$ increases of 30–56%).

With reference to aggregates 1–5, for which the TAL values were known (Table 8), it was possible to discriminate their alkali-reactivity using the TAL-based reactivity classification proposed in our previous paper: TAL < 2.8 kg Na_2_Oeq/m^3^: rapidly reactive; 2.8 ≤ TAL ≤ 5.5: moderately reactive; 5.5 < TAL ≤ 7.4: slowly reactive; TAL > 7.4: non-reactive [44].

According to this classification, aggregate 5 was classed as rapidly reactive, aggregate 1 as moderately reactive, aggregates 3 and 4 as slowly reactives and aggregate 2 as non-reactive. The classification of aggregate 2 was in contrast with its reactivity classification (Class III-S) obtained from petrographical examination (Table 1).

Comparing the Lac and TAL values reported in Table 8 for each aggregate, deleterious ASR expansion development may be predicted only for the dam concrete mix made with Portland cement and aggregate 5 (Lac>TAL). It was notewhorty that no deleterious ASR expansion was predicted for the concrete mix made with aggregate 1 (Lac<TAL), although this aggregate was characterized by a moderate alkali-reactivity according to its TAL value and was also known for its deleterious expansive behaviour in some ordinary concrete structures.

In the case of the Portland cement concrete mix made with aggregate 5, for which deleterious ASR expansion was predicteted in the long term, Equations (2) and (4) were used to calculate the maximum $Lac_{0}$ value for which no deleterious ASR expansion will develop ($maxLac_{0}=TAL-Lac_{lab}^{agg}$). A $maxLac_{0}$ value of 1.68 kg Na_2_Oeq/m^3^ was obtained, corresponding to an alkali content of cement equal to 0.84% Na_2_Oeq, for concrete mix with 200 kg/m^3^ of cement.

Alternatively, replacement of Portland cement with pozzolanic cement CEM IV/B-P ($R_{max}$ = 8.1 kg Na_2_Oeq/m^3^) in concrete made with aggregate 5 ($Lac_{0}$ = 4.4 kg Na_2_Oeq/m^3^) would yield a Lac value of 5.52 kg Na_2_Oeq/m^3^ that was much lower than the $TAL+R_{max}$ value of 10.9 kg Na_2_Oeq/m^3^ ($Lac^{*}$ value). As a result, no deleterious ASR expansion will be expected in the long-term for such a concrete mix.

This result confirmed the ability of pozzolanic cements containing natural pozzolan in counteracting ASR expansion development in concrete structures, in accordance with both the practical experience and the precautionary measures recommended by the European Report CEN/TR 16349 [45] and by RILEM [46].

It should also be emphasized that differences between laboratory expansion measurements and field behavior of concrete may often exist [47]. This should be taken into account as much as possible, eventually looking for suitable correlations, able to verify and validate the laboratory predictions.

## 5. Conclusions

Based on the results of this study, the following conclusions can be drawn:

(1) The modified alkali extraction test with saturated calcium hydroxide solution is suitable for maximizing the alkali extraction from concrete aggregates. With respect to the Italian Standard test method UNI 11417-2, the modifications consist of replacing the glass boiler with reflux by a laboratory autoclave operated at 105 °C, reducing the amount of aggregate tested from 400 to 200 g, and prolonging the test duration from 6 to 120 h. No change of the liquid/aggregate ratio (L/S = 0.6 g leachant/g dry aggregate) and the solid lime/aggregate ratio (CH/S = 5 g CH/100 g dry aggregate) was found to be necessary.
(2) With the use of the modified extraction test, the amounts of alkaline metals released from the sands investigated were found to vary from 46 to 690 mg/kg dry aggregate for sodium and from 71 to 576 mg/kg dry aggregate for potassium, corresponding to percentage releases of 0.93–2.84% for Na and 0.41–3.35% for K. These results are congruent with those available in the literature for concrete aggregates subjected to leaching tests using sodium hydroxide and potassium hydroxide as leaching media.
(3) For coarse aggregates, much lower releases of alkaline metals were found (6–11 mg/kg dry aggregate for both sodium and potassium), due to their lower specific surface area, compared to the respective sands. However, these very low releases may not be neglected in calculating the alkali release from combined aggregates (sand + coarse aggregate), in consideration of the very high content of coarse aggregates in concrete mixes. As demonstrated in this study, if the alkali release from sand is only known, it is possible to estimate the alkali release from coarse aggregate in terms of an equivalent sand content of 23 wt %.
(4) A simple model is proposed to predict the potential effect of alkali release from aggregates on deleterious ASR expansion development in long-service concrete structures. This model is based on the knowledge of four key parameters relevant to the components of the concrete mix, such as the initial alkali content of the mix used for the structure construction ($Lac_{0}$), the efficacy parameter ($R_{max}$) related to cement, the Threshold Alkali Level (TAL) of the aggregate, and the long-term alkali contribution by this aggregate to concrete mix ($Lac_{\infty }^{agg}$), the last being estimated from the results of laboratory optimized extraction tests (maximum alkali release).
(5) Application of the above model to a typical dam concrete mix leads to ASR expansion predictions that are congruent with both the field experience and the ASR prevention criteria recommended by European Technical Report CEN/TR 16349:2012 and by RILEM Specifications, thus indicating the suitability of the proposed model.

## Figures and Tables

**Table 1:** Lithological compositions of sands investigated.

Sand	Lithological Composition
S1	medium to fine grained sedimentary carbonate rocks including mono- or polycrystalline quartz, rarely showing undulatory extinction angle, flint and chalcedony
S2	sedimentary carbonatic rocks and sandstones with flint as the main alkali reactive phase
S3	arenaceous, quartzitic-feldspatic and epidote rocks, with fine flints (sometimes with a fibrous-radiate texture typical of chalcedony), mono- and polycrystalline quartz and fine-grained quartzites with a marked undulatory extinction angle
S4	similar to sand S3, except for a smaller amount of flint and a remarkable presence of carbonate rocks
S5	cataclasite, a metamorphic rock formed by mechanical fracturing on fault lines. Main constituents of this sand were feldspar particles within a strongly stressed quartz matrix, fractured feldspars, dark minerals and mica
S6	granite, an intrusive igneous rock mainly containing strained and micro-crystalline quartz, K-feldspar, plagioclase and biotite
S7	granodiorite, an intrusive igneous rock similar to sand S6, with a higher content of plagioclase and a lower content of K-feldspar than S6, containing strained and poorly crystalline quartz, biotite and hornblende

**Table 2:** Contents of sodium and potassium of aggregates investigated.

Aggregate	Na (g/kg)	K (g/kg)	Na_2_O (g/kg)	K_2_O (g/kg)	Na_2_Oeq (g/kg) *
S1	4.97	4.98	6.70	6.00	10.70
S2	13.50	10.04	18.20	12.10	26.20
S3	5.94	5.97	8.00	7.20	12.70
S4	7.27	9.63	9.80	11.60	17.50
S5	24.26	14.77	32.70	17.80	44.40
S6	22.18	43.07	29.90	51.90	64.10
S7	21.59	35.93	29.10	43.30	57.60
C1	13.00	10.00	17.52	12.05	25.47
C2	5.00	5.10	6.74	6.15	10.79

* Na_2_Oeq = Na_2_O + 0.658 K_2_O.

**Table 3:** Grain size gradation of sands subjected to leaching tests.

Grain Size	Weight Percent
4–2 mm	10
2–1 mm	20
1 mm–500 m	20
500–250 m	25
250–125 m	15
<125 m	10

**Table 4:** Grain size gradation of coarse aggregates tested.

Grain Size (mm)	Weight Percent	
	C1	C2
12–16	18	22
16–20	27	34
20–24	35	28
24–32	20	16

**Table 5:** Alkali releases from all the sands investigated under optimal test conditions.

Sand	$M_{r}\;(\mathrm{mg}\;\mathrm{Alkaline}\;\mathrm{Metal}\;\mathrm{Released}/\mathrm{kg}\;\mathrm{Dry}\;\mathrm{Aggregate})$		Percentage Release (wt %)		${M^{\prime }}_{r}\;(\mathrm{mg}\;\mathrm{Alkaline}\;\mathrm{Metal}\;\mathrm{Oxide}\;\mathrm{Released}/\mathrm{kg}\;\mathrm{Dry}\;\mathrm{Aggregate})$		
	Na	K	Na	K	Na_2_O	K_2_O	Na_2_Oeq
S1	46	71	0.93	1.43	62	86	118
S2	96	41	0.71	0.41	129	49	162
S3	172	88	2.90	1.47	232	106	302
S4	68	66	0.94	0.69	92	80	144
S5	690	495	2.84	3.35	930	597	1323
S6	287	431	1.29	1.00	387	519	729
S7	549	576	2.54	1.60	740	694	1198

**Table 6:** Alkali releases from coarse aggregates investigated.

Coarse Aggregate	$M_{r}\;(\mathrm{mg}\;\mathrm{Metal}\;\mathrm{Released}/\mathrm{kg}\;\mathrm{Dry}\;\mathrm{Aggregate})$		Metal Release (wt %)		${M^{\prime }}_{r}\;(\mathrm{mg}\;\mathrm{Alkali}\;\mathrm{Released}/\mathrm{kg}\;\mathrm{Dry}\;\mathrm{Aggregate})$		
	Na	K	Na	K	Na_2_O	K_2_O	Na_2_Oeq
C1	6	11	0.05	0.11	8	13	17
C2	11	8	0.22	0.16	15	10	21

**Table 7:** Estimated $\Delta X_{S}\%$ values for the S1-C1 and S2-C2 aggregate combinations.

Combined Aggregate	$X_{S}$	Release (mg Na/kg Combined Aggregate)		${X^{\prime }}_{S}$	$\Delta X_{S}\%$	Release (mg K/kg Combined Aggregate)		${X^{\prime }}_{S}$	$\Delta X_{S}\%$
		$M_{r\;Comb}$	$M_{r\;Comb}^{*}$			$M_{r\;Comb}$	$M_{r\;Comb}^{*}$		
S1-C1	0.35	20	19.8	0.43	24.2	32	30.6	0.43	28.8
S2-C2	0.35	41	41.3	0.42	21.3	20	17.7	0.48	36.2
S1-C1	0.45	24	25.4	0.52	15.9	38	39.3	0.54	18.9
S2-C2	0.45	49	53.1	0.51	14.0	23	22.7	0.56	23.8

**Table 8:** Values of $TAL,\;Lac_{lab}^{agg}$ and Lac for ASR prediction in Portland cement concrete dams.

Aggregate	TAL (kg Na_2_Oeq/m^3^)	$Lac_{lab}^{agg}$ (kg Na_2_Oeq/m^3^)	Lac (kg Na_2_Oeq/m^3^)
1	3.9	0.10	2.10
2	8.2	0.14	2.14
3	7.2	0.26	2.26
4	6.0	0.12	2.12
5	2.8	1.12	3.12
6	n.a.	0.62	-
7	n.a.	1.01	-

n.a. = not available.
